# Factors Associated With Acute Kidney Injury in Patients Undergoing Transcatheter Aortic Valve Implantation: A Systematic Review and Meta-Analysis

**DOI:** 10.7759/cureus.45131

**Published:** 2023-09-12

**Authors:** Karima Benaicha, Basel Aldroubi, Paras Yousuf, Rabinder Nath, FNU Saveeta, FNU Kanwal, Tehreem Fatima, Shamsha Hirani

**Affiliations:** 1 Internal Medicine, University Hospital Isaad Hassani Beni Messous, Algiers, DZA; 2 Medical College, Tishreen University, Latakia, SYR; 3 Emergency Medicine, Jinnah Postgraduate Medical Centre, Karachi, PAK; 4 Medical College, Ghulam Muhammad Mahar, Sukkur, PAK; 5 Internal Medicine, People's University of Medical and Health Sciences, Nawabshah, PAK; 6 Medical College, Chandka Medical College, Larkana, PAK; 7 Internal Medicine, United Medical and Dental College, Creek General Hospital, Karachi, PAK; 8 Cardiology, Baqai Hospital, Karachi, PAK

**Keywords:** systematic review and meta-analysis, post-operative, factors, transcatheter aortic valve implantation (tavi), acute kidney injury

## Abstract

The aim of this meta-analysis is to assess the effect of different independent predictors on acute kidney injury (AKI) after transcatheter aortic valve implantation (TAVI). This meta-analysis adhered to the guidelines of the Preferred Reporting Items for Systematic Reviews and Meta-Analyses (PRISMA). A comprehensive database search was conducted using PubMed, Web of Science, and Scopus for the period from January 1, 2015, to August 15, 2023. The following key terms were employed: "transcatheter aortic valve implantation" OR "transcatheter aortic valve replacement" AND "acute kidney injury" OR "acute renal failure." Our search was limited to studies published exclusively in the English language. The statistical analysis was conducted using RevMan version 5.4.1 (The Cochrane Collaboration). Estimates were presented as odds ratio (OR) with 95% confidence interval (CI) for categorical variables, while continuous variables were reported as mean difference (MD) with 95% CI. A total of 19 studies met the selection criteria and were included in the meta-analysis. The pooled incidence of AKI was reported as 20% (95% CI: 18-20%). Factors significantly associated with post-TAVI AKI encompass hypertension, chronic kidney disease (CKD), low estimated glomerular filtration rate (eGFR), high baseline creatinine levels, peripheral vascular disease (PVD), Society of Thoracic Surgeons (STS) score, European System for Cardiac Operative Risk Evaluation (EUROscore) II, and the transfemoral surgical approach.

## Introduction and background

Transcatheter aortic valve implantation (TAVI) is a contemporary and extensively researched approach for addressing symptomatic individuals afflicted by severe aortic stenosis (AS) [1]. A multitude of investigations have substantiated its effectiveness, manifesting advantages in terms of reduced mortality rates and enhanced quality of life [2,3]. Nonetheless, the TAVI procedure is not without significant hazards. Despite advancements in technique and experience, there remains a notable occurrence of peri- and post-procedural complications [4]. One such complication is acute kidney injury (AKI), which is acknowledged for its severe clinical consequences within the TAVI context [5]. Causative factors implicated in AKI development post-TAVI encompass compromised baseline kidney function, heightened contrast agent usage, procedural hemodynamic instability, substantial bleeding during the procedure, embolization of materials distal to the site, and application of potentially nephrotoxic agents [6,7].

TAVI is typically performed on elderly and high-risk patients, who often present with both a high prevalence of chronic kidney disease (CKD) and heart failure [8]. Additionally, TAVI entails the utilization of contrast agents and the manipulation of large catheters within the aorta, elevating the vulnerability to complications like contrast-induced nephropathy and the dislodgment of atherosclerotic particles into the renal vascular domain [8]. Periods of hypotension, such as those occurring during aortic balloon valvuloplasty, rapid pacing, and valve delivery, can further contribute to renal impairment [8]. AKI in TAVI patients is believed to arise from a combination of prerenal azotemia and direct nephrotoxic effects, culminating in renal ischemia and acute tubular necrosis [4].

Although the majority of TAVI-related AKI cases are mild, the impact is particularly pronounced in older, more frail patients with established or potential underlying kidney dysfunction. This risk is compounded in scenarios where the patient necessitates additional common angiographic procedures [1]. TAVI patients constitute a distinctive demographic characterized by intricate comorbidities and frequently advanced age [4]. This meta-analysis encapsulates recent research and evidence surrounding potential risk factors linked to heightened post-TAVI AKI occurrence. By comparing findings across existing literature, this analysis contributes to refining risk assessment for individuals undergoing TAVI procedures.

The aim of this meta-analysis is to assess the effect of different independent predictors on AKI after TAVI. By understanding the impact of different factors on AKI after TAVI, it will help physicians to identify patients at high risk of AKI; thus, early management can prevent the development of AKI in this population.

## Review

Methodology

This meta-analysis adhered to the guidelines of the Preferred Reporting Items for Systematic Reviews and Meta-Analyses (PRISMA).

Search Strategy and Study Selection

A comprehensive database search was conducted using PubMed, Web of Science, and Scopus for the period from January 1, 2015, to August 15, 2023. The following key terms were employed: "transcatheter aortic valve implantation" OR "transcatheter aortic valve replacement" AND "acute kidney injury" OR "acute renal failure." Our search was limited to studies published exclusively in the English language. All articles centered on risk factors associated with post-TAVI AKI. The reference lists of all included studies were also manually reviewed.

All eligible records were imported into EndNote X9. After eliminating duplicates, initial screening was performed based on abstracts and titles, followed by full-text screening using predefined inclusion and exclusion criteria. We encompassed studies in which participants underwent TAVI and studies that evaluated factors linked to post-operative AKI. Our inclusion encompassed studies regardless of the criteria utilized to diagnose AKI. We excluded reviews, editorials, meta-analyses, and expert opinions.

Data Extraction and Quality Assessment

Two authors independently extracted the following data from the included studies: first author, publication year, study setting, sample size, number of patients who developed AKI, criteria employed to diagnose AKI, and patients' characteristics. The quality assessment of the included studies was carried out using the Newcastle-Ottawa Scale. This scale allocates stars based on three pivotal criteria: selection of study groups, comparability of groups, and ascertainment of exposure/outcome. The scale facilitates the identification of bias risk in observational studies, offering a standardized evaluation of study quality across diverse research articles.

Data Analysis

Estimates were presented as odds ratio (OR) with 95% confidence interval (CIs) for categorical variables, while continuous variables were reported as mean difference (MD) with 95% CI. Study heterogeneity was assessed using the I^2^ statistic. A random-effects model was applied regardless of heterogeneity to address variations due to study settings, study populations, and AKI diagnostic criteria. All p-values were two-sided, and significance for heterogeneity was considered at a p-value <0.05. The statistical analysis was conducted using RevMan version 5.4.1 (The Cochrane Collaboration).

Results

**Figure 1:**
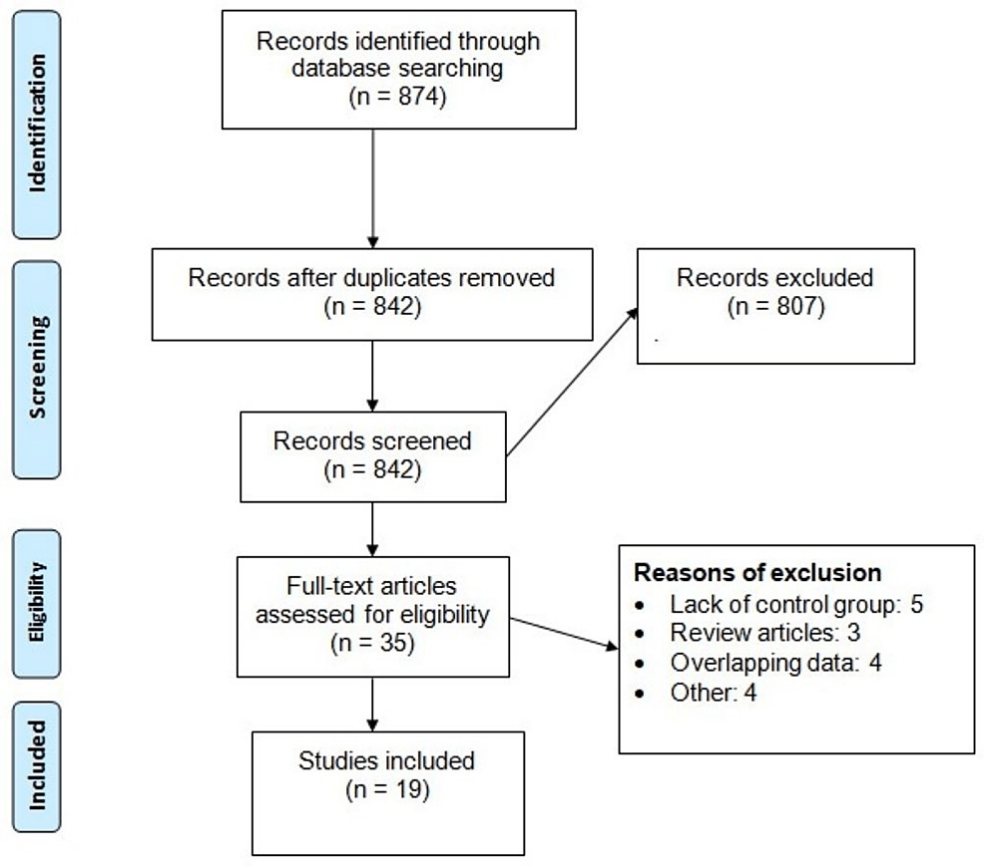
PRISMA flowchart. PRISMA: Preferred Reporting Items for Systematic Reviews and Meta-Analyses.

Figure 1 shows the PRISMA flowchart of study selection. Database searching yielded 874 records. After removing duplicates, we initially screened 842 records using titles and abstracts. A total of 35 studies were eligible for detailed evaluation of inclusion and exclusion criteria. Finally, 19 studies met the selection criteria and were included in the meta-analysis. Table 1 shows the characteristics of included studies. The pooled incidence of AKI was reported 20% (95% CI: 18-22%). Table 2 presents the quality assessment of all included studies.

**Table 1:** Characteristics of included studies. AKI: acute kidney injury; VARC-2: Valve Academic Research Consortium-2; RIFLE: Risk, Injury, Failure, Loss of kidney function, and End-stage kidney disease; KDIGO: Kidney Disease Improving Global Outcomes; NR: not reported.

Author name	Year	Region	Defining AKI	Sample size (N)	AKI (n)
Abbas et al. [9]	2021	Pakistan	NR	173,760	20,045
Alhamad et al. [10]	2023	Saudi Arabia	NR	344	60
Arsalan et al. [11]	2016	United States	VARC-2 criteria	230	144
Crimi et al. [12]	2022	Italy	VARC-2 criteria	2,621	452
Crowhurst et al. [13]	2021	France	VARC-2 criteria	574	48
Elmariah et al. [14]	2016	Australia	RIFLE	209	82
Kandathil et al. [15]	2018	United States	KDIGO	106	20
Koifman et al. [16]	2016	United States	VARC-2 criteria	44	9
Konigstein et al. [17]	2015	Israel	VARC-2 criteria	422	66
Kumar et al. [18]	2019	United States	NR	39,898	7,461
Meneguz-Moreno et al. [19]	2017	Brazil	VARC-2 criteria	221	52
Merchant et al. [20]	2019	United States	KDIGO	116	20
Miura et al. [21]	2019	Japan	VARC-2 criteria	81	7
Peillex et al. [22]	2016	Israel	KDIGO	217	49
Rosendael et al. [23]	2015	Netherlands	VARC-2 criteria	210	51
Shishikura et al. [24]	2018	Australia	VARC-2 criteria	278	92
Sudarsky et al. [25]	2021	Israel	KDIGO	210	38
Thongprayoon et al. [26]	2016	United States	KDIGO	386	106
Zahid et al. [27]	2021	United States	NR	216,023	27,871

**Table 2:** Quality assessment. Selection score: Out of three. Exposure: Out of two. Outcome: Out of four.

Author name	Selection	Exposure	Outcome	Overall
Abbas et al. [9]	3	1	2	Fair
Alhamad et al. [10]	3	1	2	Fair
Arsalan et al. [11]	2	2	3	Good
Crimi et al. [12]	3	2	3	Good
Crowhurst et al. [13]	3	2	3	Good
Elmariah et al. [14]	3	2	4	Good
Kandathil et al. [15]	3	2	3	Good
Koifman et al. [16]	3	2	3	Good
Konigstein et al. [17]	2	1	3	Fair
Kumar et al. [18]	3	1	2	Fair
Meneguz-Moreno et al. [19]	2	2	3	Good
Merchant et al. [20]	3	2	2	Good
Miura et al. [21]	3	2	2	Good
Peillex et al. [22]	3	1	3	Good
Rosendael et al. [23]	3	2	2	Good
Shishikura et al. [24]	3	2	3	Good
Sudarsky et al. [25]	2	2	3	Good
Thongprayoon et al. [26]	3	2	3	Good
Zahid et al. [27]	3	2	3	Good

Factors Associated With AKI

Table 3 discusses factors associated with AKI post-TAVI. As shown in Table 2, there is no significant difference in age, left ventricular ejection fraction (LVEF), and body mass index (BMI) between patients who developed AKI and patients who did not develop AKI. However, patients who developed AKI had significantly higher creatinine (MD: 0.24, 95% CI: 0.19-0.29), higher EUROscore II (European System for Cardiac Operative Risk Evaluation) (MD: 1.25, 95% CI: 0.64-1.86), and higher Society of Thoracic Surgeons (STS) score (MD: 1.57, 95% CI: 0.81-2.33) compared to patients without AKI. Additionally, patients who developed AKI had a significantly lower baseline glomerular filtration rate (GFR) compared to patients who did not develop AKI (MD: -10.37, 95% CI: -11.81 to -8.92).

**Table 3:** Relationship of continuous variables with AKI. MD>1 shows a higher mean of variables in AKI patients compared to non-AKI, while MD<1 shows a lower mean of variables in non-AKI patients compared to AKI. GFR: glomerular filtration rate; BMI: body mass index; STS: Society of Thoracic Surgeons; LVEF: left ventricular ejection fraction; EUROscore: European System for Cardiac Operative Risk Evaluation; MD: mean difference; CI: confidence interval. ^*^Significant at p-value <0.05.

Variables	MD (95% CI)	p-value	I^2^
Age	0.23 (-0.29 to 0.76)	0.38	91%
Creatinine*	0.24 (0.19 to 0.29)	0.001	77%
GFR*	-10.37 (-11.81 to -8.92)	0.001	7%
BMI	-0.27 (-1.12 to 0.58)	0.53	83%
LVEF	-1.32 (-3.42 to 0.78)	0.22	88%
EUROscore II*	1.25 (0.64 to 1.86)	0.001	18%
STS score*	1.57 (0.81 to 2.33)	0.001	77%

Table 4 displays the associations between various factors and AKI. Gender, diabetes, dyslipidemia, myocardial infarction (MI), and New York Heart Association (NYHA) Functional Classification were not found to be significantly associated with AKI. However, patients with AKI exhibited significantly higher odds of CKD compared to their counterparts (OR: 3.68, 95% CI: 2.64-5.13). Furthermore, the odds of hypertension and peripheral vascular disease (PVD) were also significantly higher in patients who experienced AKI compared to those who did not (p<0.05). Notably, patients who underwent TAVI through the transfemoral (TF) approach demonstrated a significantly lower incidence of AKI (OR: 0.70, 95% CI: 0.59-0.82).

**Table 4:** Relationship of categorical variables with AKI. OR>1 shows a higher proportion of that variable in AKI patients compared to non-AKI, while OR<1 shows a lower proportion of that variable in AKI patients compared to non-AKI. CKD: chronic kidney disease; PVD: peripheral vascular disease; MI: myocardial infarction; NYHA: New York Heart Association; OR: odds ratio; CI: confidence interval; TF: transfemoral. ^*^Significant at p-value <0.05.

Variables	OR (95% CI)	p-value	I^2^
Gender (male)	1.07 (0.95-1.20)	0.26	64%
Hypertension*	1.03 (0.99-1.06)	0.049	87%
Diabetes	1.05 (0.87-1.27)	0.61	94%
CKD*	3.68 (2.64-5.13)	0.001	99%
PVD*	1.22 (1.12-1.32)	0.001	69%
Dyslipidemia	0.98 (0.76-1.27)	0.88	33%
MI	1.13 (0.98-1.31)	0.09	0%
NYHA (III or IV)	0.93 (0.76-1.14)	0.48	72%
TF access*	0.70 (0.59-0.82)	0.001	76%

Discussion

The current meta-analysis evaluates the risk factors linked to AKI in patients undergoing TAVI. Factors significantly associated with post-TAVI AKI encompass hypertension, CKD, low estimated GFR (eGFR), high baseline creatinine levels, PVD, SSC score, EUROscore II, and the TF surgical approach. Notably, the meta-analysis underscores a notable, independent risk associated with a previous history of CKD.

This finding aligns with studies by Wang et al. [28] and Wessely et al. [29], both of which report a correlation between AKI and baseline renal function. Moreover, our analysis reveals substantially elevated creatinine and diminished eGFR levels at baseline among patients who developed AKI following TAVI. Such observations signify compromised kidney function; decreased eGFR implies diminished filtration capacity possibly stemming from CKD, AKI, or underlying conditions such as diabetes and hypertension [30]. Similarly, heightened creatinine levels, a metabolic byproduct, indicate reduced kidney clearance, suggesting impaired filtration [31]. Elevated creatinine often accompanies decreased kidney function, reflecting impaired filtration. Both eGFR and creatinine serve as pivotal markers for assessing kidney health and gauging the severity of potential dysfunction [32]. It is pivotal to recognize that, while informative, these markers necessitate a comprehensive evaluation incorporating medical history, clinical symptoms, and diagnostic tests to comprehensively assess kidney health and ascertain causal factors.

Our analysis indicates that previous occurrences of PVD and hypertension stand as risk factors for post-TAVI AKI. Wang et al.'s multivariable regression [28] further corroborates PVD's risk in post-TAVI AKI. PVD-related complications impair inherent kidney repair mechanisms, rendering the kidney susceptible to reduced blood flow (hypoperfusion). These complications affect vessels of varying sizes, undermining the kidney's natural repair capacity and rendering it prone to inadequate blood supply [33]. A history of hypertension may reduce resistance in afferent arterioles, potentially compromising renal autoregulation [34]. These conditions collectively elevate the chances of renal hypoperfusion and ensuing AKI post-TAVI.

Our meta-analysis highlights a decreased AKI risk associated with TF access in TAVI. A recent observational meta-analysis identified a higher mortality risk in transapical TAVI compared to TF access [35]. Moreover, TF access demonstrated lower occurrences of left ventricular dysfunction and myocardial injury than transapical access [36]. These findings suggest that opting for TF access might result in milder effects on kidney function.

A limitation of this meta-analysis is substantial heterogeneity, likely stemming from diverse AKI diagnostic criteria and study settings. Additionally, in clinical practice, TAVI procedures predominantly employ TF access, reserving the transapical approach for infeasible cases. Notably, most transapical TAVI patients have PVD. A recent study confirmed pre-existing PVD as an independent AKI risk factor post-TAVI. While our findings also indicate an increased AKI risk with transapical TAVI, it is crucial to note that the transapical method itself might not adversely affect kidneys; antecedent PVD, rather than the method, amplifies AKI risk. To comprehensively grasp the transapical access and post-TAVI AKI correlation, contrasting AKI occurrences in transapical TAVI patients without PVD versus those with PVD is imperative.

## Conclusions

In conclusion, this meta-analysis offers comprehensive insights into the risk factors associated with AKI in patients undergoing TAVI. Through the rigorous evaluation of a substantial dataset, the analysis has illuminated critical factors contributing to AKI occurrence post-TAVI. Notably, the pooled incidence of AKI was determined to be 20% (95% CI: 18-22%), underscoring the significance of this complication. Factors significantly associated with post-TAVI AKI, including hypertension, PVD, high creatinine, low GFR, CKD, EUROscore II, STS score, and TF approach. Further research addressing the limitations of heterogeneity and refining our understanding of the interplay between PVD and surgical approach will continue to enhance our comprehension of AKI dynamics in this patient population.
